# Th1, Th2, and Th17 cells and their corresponding cytokines are associated with anxiety, depression, and cognitive impairment in elderly gastric cancer patients

**DOI:** 10.3389/fsurg.2022.996680

**Published:** 2022-10-25

**Authors:** Yanxia Zhou, Ke Yu

**Affiliations:** ^1^ Nursing Department, Chenzhou First People’s Hospital of Hunan Province, Chenzhou, China; ^2^ Operation Room, Changsha Hospital for Maternal / Child Health Care Affiliated to Hunan Normal University, Changsha, China

**Keywords:** elderly gastric cancer patients, th cells, anxiety, depression, cognitive impairment

## Abstract

**Objective:**

T helper (Th) cells modulate the stress response, oxidative stress, and neuroinflammation to mediate anxiety, depression, and cognitive impairment. This study intended to explore the association between Th cells and anxiety, depression, and cognitive impairment in elderly gastric cancer patients.

**Methods:**

Totally, 176 elderly gastric cancer patients were enrolled in this study. Peripheral blood samples were collected. Th1, Th2, and Th17 cells were detected by flow cytometry; their corresponding cytokines were examined by ELISA. The Hospital Anxiety and Depression Scale (HADS) and Mini-Mental State Examination (MMSE) were assessed.

**Results:**

In total, 42.0%, 33.0%, and 19.9% of elderly gastric cancer patients presented anxiety, depression, and cognitive impairment, respectively. Th1 (*P* = 0.016), Th17 (*P* = 0.009), and IL-17A (*P* = 0.001) were positively associated with the HADS-A score. Th17 (*P* = 0.003) and IL-17A (*P* = 0.009) levels were increased in patients with anxiety compared with those without anxiety. Concurrently, a positive association was observed for Th1 (*P* = 0.027), Th17 (*P* = 0.014), and IFN-γ (*P* = 0.049) with the HADS-D score. Th1 (*P* = 0.017) and Th17 (*P* = 0.049) levels were increased in patients with depression than in those without depression. Moreover, Th1 (*P* = 0.003), Th17 (*P* < 0.001), IFN-γ (*P* = 0.014), and IL-17A (*P* < 0.001) were inversely related to MMSE scores, but only Th17 (*P* < 0.001) and IL-17A (*P* < 0.001) were increased in patients with cognitive impairment compared with those without cognitive impairment.

**Conclusion:**

Th1 and Th17 cells reflect anxiety, depression, and cognitive impairment risk to a certain extent in elderly gastric cancer patients, implying their involvement in the pathology of the abovementioned psychological and cognitive issues. However, further validation is needed.

## Introduction

Gastric cancer is the fifth most commonly diagnosed cancer and the fourth leading cause of cancer death globally, representing a severe threat to public health (1, 2). Notably, age is one of the risk factors for gastric cancer, indicating that elderly populations are more likely to suffer from this carcinoma (3). At present, certain progress has been made in the treatment of elderly gastric cancer patients, including surgery, chemotherapy, targeted therapy, immunotherapy, etc. (4–6). Nevertheless, due to the high mortality rate and the occurrence of postoperative complications (such as gastric perforation, leakage, pancreatic fistula, gastrointestinal bleeding, etc.) (7–10), anxiety and depression are prevalent in elderly gastric cancer patients (11). Moreover, aging issues also make elderly patients more likely to develop cognitive impairment (12, 13). Thus, exploring biomarkers to monitor psychological status and cognitive functions and improving the clinical outcomes of elderly gastric cancer patients is necessary.

Previous studies have reported that CD4^+^ T cells [T helper (Th) cells] may participate in the pathology and progression of anxiety, depression, and cognitive impairment (14–16). A study revealed that dramatic mitochondrial fission in CD4^+^ T cells contributes to anxiety and depression in mice (14). In addition, the upregulation of IL-17A contributes to the accumulation of mild stress, which further causes depressive symptoms in mice (16). Regarding cognitive impairment, a study claims that increases in Th17 and interleukin (IL)-17A reduces nitric oxide production, thereby facilitating cognitive dysfunction in mice fed with excess dietary salt (15). Clinically, the relationship between Th-cell-secreted cytokines and anxiety, depression, and cognitive impairment in cancer patients has also been revealed by several previous studies (17, 18). For instance, elevated tumor necrosis factor-α and IL-17 indicate enhanced anxiety and depression risks in non-small cell lung cancer (NSCLC) patients (17). Moreover, IL-4 is increased in breast cancer patients with cognitive impairment (18). Based on the above evidence, it could be hypothesized that CD4^+^ T cells might be linked to anxiety, depression, and cognitive impairment in elderly gastric cancer patients. Unfortunately, no study has reported this association.

Accordingly, this study intended to explore the association of Th1, Th2, and Th17 cells as well as their corresponding cytokines [including interferon (IFN)-γ, IL-4, and IL-17A] with anxiety, depression, and cognitive impairment in elderly gastric cancer patients.

## Methods

### Patients

This two-center, prospective study consecutively included 176 elderly patients with resectable gastric cancer who received surgical resection from November 2019 to January 2022. The inclusion criteria were as follows: (i) pathologically confirmed gastric cancer; (ii) aged ≥60 years; (iii) received surgical resection; (iv) willing to receive peripheral blood (PB) collection for study use; and (v) able to complete the Mini-Mental State Examination (MMSE >20 at 3 months after surgery). The exclusion criteria were as follows: (i) metastatic gastric cancer; (ii) a previous history of diagnosed anxiety or depression; (iii) concomitant with other primary cancers or hematological malignancies; and (iv) a history of diseases affecting cognitive functions, such as stroke, schizophrenia, and Alzheimer's disease. The study was approved by the Ethics Committee. Each patient provided informed consent.

### Collection of data and sample

The clinical characteristics of elderly gastric cancer patients, including demographics, disease history, cancer-related features, and treatment information, were documented after recruitment. PB samples were collected at 3 months after surgery (±2 weeks) to detect the proportions of Th cells and the levels of their corresponding cytokines (IFN-γ, IL-4, and IL-17A).

### Sample processing

After the separation of CD4^+^ T cells from PB samples using Dynabeads™ FlowComp™ CD4 kit (ThermoFisher, USA), the proportions of T helper 1 (Th1) cells, T helper 2 (Th2) cells, and T helper 17 (Th17) cells in CD4^+^ T cells were examined by flow cytometry (FCM) using the Human Th1/Th2/Th17 Phenotyping Kit (BD, USA) *via* the recommended procedures of the experiments. Briefly, phorbol myristate acetate (50 ng/ml, BD, USA) plus ionomycin (1 μg/ml, BD, USA) was used to stimulate cells for 5 h, and the cells were transferred into flow-quiet tubes. Next, the cells were suspended in 50 µl of BD Perm/Wash™ buffer with 20 µl/tube cocktail and then incubated for 30 min in the dark for staining. The utilized antibody cocktail contained human CD4 PerCP-Cy5.5 (clone: SK3), human IL-17A PE (clone: N49-653), human IFN-γ FITC (clone: B27), and human IL-4 APC (clone: MP4-25D2). Next, the stained cells were analyzed by flow cytometric analysis using a FC-500 flow cytometer (Beckman Coulter, USA). The gating strategy was as follows: CD4^+^ IFN-γ^+^ for Th1 cells; CD4^+^ IL-4^+^ for Th2 cells; and CD4^+^ IL-17A^+^ for Th17 cells.

The serum samples were isolated by centrifugation at 3,500 r/min for 10 min, and then the cytokine levels in serum were examined by enzyme-linked immunosorbent assay (ELISA) using Human Quantikine ELISA Kits (R/D Systems, USA) in accordance with the instructions. In brief, 100 µl of assay diluent and 50 µl of standards, control, or sample were added into each well and then incubated for 2 h. Next, the well was washed 4 times, and 100 µl of the conjugate was added. The sample was incubated for 1 h and washed 4 times. Subsequently, 200 µl of substrate solution was added and incubated at room temperature for 30 min in the dark. Then, 50 µl of stop solution was added, and the absorbance was immediately read at 450 nm. The concentrations of cytokines were finally calculated based on the standard curve.

### Assessment

The Hospital Anxiety and Depression Scale (HADS) and MMSE were scored at the same time as the PB samples obtained. HADS for anxiety (HADS-A) and HADS for depression (HADS-D) were used for anxiety and depression evaluation, respectively, with results based on the following scale: no, 0–7; mild, 8–10; moderate, 11–14, severe, 15–21 (19). The MMSE score was used for cognitive impairment evaluation as follows: no, 27–30; mild, 21–26; moderate, 10–20; severe, 0–9 (20).

### Statistics

SPSS (version 22.0, IBM Corp., USA) was applied for analyses. GraphPad Prism (version 7.01, GraphPad Software Inc., USA) was applied to plot figures. Normality was assessed using the Kolmogorov‒Smirnov test. Normally distributed continuous variables are displayed as the mean ± standard deviation (SD). Skewed distributed continuous variables are displayed as the median and interquartile range (IQR). Categorized variables are displayed as counts (percentages). The normally distributed continuous variables (HADS-A score, HADS-D score, and MMSE score) among patients with different treatment information were analyzed using Student's *t* test or one-way analysis of variance (ANOVA). Skewed distributed continuous variables (Th cells and cytokines) among patients with different treatment information were analyzed using Wilcoxon rank-sum test or Kruskal‒Wallis H rank-sum test. Categorized variables (anxiety rate, depression rate, and cognitive impairment rate) among patients with different treatment information were analyzed using Chi-square test or linear by linear test. Associations between continuous variables were analyzed using Spearman's rank correlation test. The levels of Th cells and cytokines between patients with or without anxiety, depression, and cognitive impairment were determined using the Wilcoxon rank-sum test. The comparison between elderly gastric cancer patients and elderly normal individuals was assessed using Student's *t* test, the Mann‒Whitney U test, and the chi-square test, as appropriate. In addition, factors related to anxiety, depression, and cognitive impairment were evaluated using a multivariate logistic regression model (all factors were included and then analyzed *via* the step-forward method). *P *< 0.05 was considered significant.

## Results

### Clinical features

The included elderly gastric cancer patients had a mean age of 68.7 ± 5.6 years. In total, 64 (36.4%) females and 112 (63.6%) males were included in this study. Regarding the disease history, there were 92 (52.3%), 52 (29.5%), and 32 (18.2%) patients with a history of hypertension, hyperlipidemia, and diabetes, respectively. Additionally, 16 (9.1%), 50 (28.4%), and 110 (62.5%) patients had tumor-node-metastasis stage I, II, and III disease, respectively. Concerning the treatment information, 110 (62.5%) patients received neoadjuvant chemotherapy. In addition, 8 (4.5%), 161 (91.5%), and 7 (4.0%) patients underwent endoscopic mucosal resection/endoscopic submucosal dissection (EMR/ESD), gastrectomy by open surgery, and gastrectomy by laparoscope, respectively. Seventy (39.8%) patients experienced postoperative complications. Furthermore, 160 (90.9%) patients received adjuvant chemotherapy. Specific clinical information on elderly gastric cancer patients is listed in Table 1.

**Table 1:** Clinical characteristics.

Items	Elderly gastric cancer patients (*N* = 176)
**Demographics**	
Age (years), mean ± SD	68.7 ± 5.6
Gender, no. (%)	
Female	64 (36.4)
Male	112 (63.6)
Current smoker, no. (%)	66 (37.5)
Current drinker, no. (%)	59 (33.5)
Education level, no. (%)	
Primary school or below	44 (25.0)
Middle or high school	88 (50.0)
Undergraduate or above	44 (25.0)
Marital status, no. (%)	
Married	103 (58.5)
Single/divorced/widowed	73 (41.5)
Residential location, no. (%)	
Rural	26 (14.8)
Urban	150 (85.2)
**Disease history**	
History of hypertension, no. (%)	92 (52.3)
History of hyperlipidemia, no. (%)	52 (29.5)
History of diabetes, no. (%)	32 (18.2)
**Cancer-related features**	
Helicobacter pylori infection, no. (%)	
Negative	100 (56.8)
Positive	76 (43.2)
Tumor location, no. (%)	
Cardia	44 (25.0)
Gastric body	65 (36.9)
Gastric antrum	67 (38.1)
Tumor size (cm), median (IQR)	3.0 (2.5-4.0)
Differentiation, no. (%)	
Well	32 (18.2)
Moderate	95 (54.0)
Poor	49 (27.8)
TNM stage, no. (%)	
Stage I	16 (9.1)
Stage II	50 (28.4)
Stage III	110 (62.5)
**Treatment information**	
Neoadjuvant chemotherapy, no. (%)	110 (62.5)
Surgery type, no. (%)	
EMR/ESD	8 (4.5)
Gastrectomy by open surgery	161 (91.5)
Gastrectomy by laparoscope	7 (4.0)
Postoperative complication, no. (%)	70 (39.8)
Adjuvant chemotherapy, no. (%)	160 (90.9)

SD, standard deviation; IQR, interquartile range; TNM, tumor-node-metastasis; EMR, endoscopic mucosal resection; ESD, endoscopic submucosal dissection.

### Anxiety, depression, cognitive impairment, Th cells, and cytokines

The mean HADS-A score in elderly gastric cancer patients was 8.2 ± 2.8, and 74 (42.0%) patients had anxiety. In addition, the mean HADS-D score was 7.5 ± 2.6, and 58 (33.0%) patients had depression. Moreover, their mean value of the MMSE score was 27.5 ± 1.6, and 35 (19.9%) patients had cognitive impairment (Table 2).

**Table 2:** Anxiety, depression, and cognitive impairment.

Items	Elderly gastric cancer patients (*N* = 176)
HADS-A score, mean ± SD	8.2 ± 2.8
Anxiety rate, no. (%)	74 (42.0)
Anxiety severity, no. (%)	
No	102 (58.0)
Mild	33 (18.8)
Moderate	36 (20.5)
Severe	5 (2.8)
HADS-D score, mean ± SD	7.5 ± 2.6
Depression rate, no. (%)	58 (33.0)
Depression severity, no. (%)	
No	118 (67.0)
Mild	28 (15.9)
Moderate	27 (15.3)
Severe	3 (1.7)
MMSE score, mean ± SD	27.5 ± 1.6
Cognitive impairment rate, no. (%)	35 (19.9)
Cognitive impairment severity, no. (%)	
No	141 (80.1)
Mild	35 (19.9)
Moderate	0 (0.0)
Severe	0 (0.0)

HADS-A, Hospital Anxiety and Depression Scale-Anxiety; SD, standard deviation; HADS-D, Hospital Anxiety and Depression Scale-Depression; MMSE, Mini-Mental State Examination.

The median (IQR, range) values of Th1, Th2, and Th17 cells were 15.0 (11.5–18.6, 7.7–30.9) %, 10.4 (8.6–13.1, 5.6–24.0) %, and 2.3 (1.3–3.4, 0.5–6.4) %, respectively. Regarding cytokines, the median (IQR, range) values of IFN-γ, IL-4, and IL-17A were 2.3 (1.7–3.3, 1.2–6.1) pg/ml, 30.9 (24.4–49.6, 17.7–91.3) pg/ml, and 43.3 (36.6–55.2, 28.2–105.7) pg/ml, respectively (Table 3).

**Table 3:** Information of Th cells and cytokines.

Items	Median	IQR	Range
Th1 cells (%) (/CD4^+^)	15.0	11.5–18.6	7.7–30.9
Th2 cells (%) (/CD4^+^)	10.4	8.6–13.1	5.6–24.0
Th17 cells (%) (/CD4^+^)	2.3	1.3–3.4	0.5–6.4
IFN-γ (pg/ml)	2.3	1.7–3.3	1.2–6.1
IL-4 (pg/ml)	30.9	24.4–49.6	17.7–91.3
IL-17A (pg/ml)	43.3	36.6–55.2	28.2–105.7

Th, T helper; IFN, interferon; IL, interleukin; IQR, interquartile range.

### Relationship of Th cells and their corresponding cytokines with anxiety

**Figure 1:**
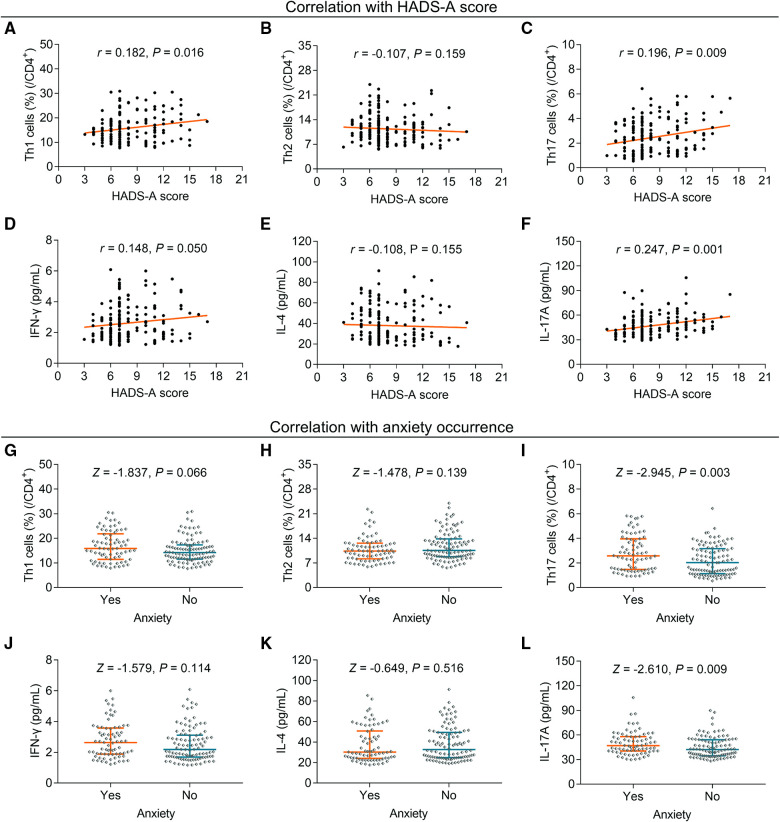
The association between Th1, Th2, and Th17 cells and anxiety in elderly gastric cancer patients. The relationship between Th1 (**A**), Th2 (**B**), Th17 cells (**C**), IFN-γ (**D**), IL-4 (**E**), and IL-17A (**F**) and HADS-A score. The relationship between Th1 (**G**), Th2 (**H**), Th17 cells (**I**), IFN-γ (**J**), IL-4 (**K**), and IL-17A (**L**) and anxiety occurrence in elderly gastric cancer patients. Statistical methods: associations between continuous variables were analyzed using Spearman's rank correlation test; the levels of Th cells and cytokines between patients with or without anxiety, depression, and cognitive impairment were determined using the Wilcoxon rank-sum test.

Th1 cells (*P* = 0.016) (Figure 1A), but not Th2 cells (*P* = 0.159) (Figure 1B), were positively associated with the HADS-A score; meanwhile, a positive relationship was found between Th17 cells and the HADS-A score (*P* = 0.009) (Figure 1C). IFN-γ (*P* = 0.050) (Figure 1D) and IL-4 (*P* = 0.155) (Figure 1E) were not associated with the HADS-A score, but IL-17A was positively associated with the HADS-A score (*P* = 0.001) (Figure 1F) in elderly gastric cancer patients.

Further analysis revealed that Th1 cells (*P* = 0.066) (Figure 1G) and Th2 cells (*P* = 0.139) (Figure 1H) did not differ between elderly gastric cancer patients with and without anxiety, whereas Th17 cells (*P* = 0.003) (Figure 1I) were increased in patients with anxiety compared to those without anxiety. IFN-γ (*P* = 0.114) (Figure 1J) and IL-4 (*P* = 0.516) (Figure 1K) levels did not differ between patients with and without anxiety, whereas IL-17A was increased in patients with anxiety compared with those without anxiety (*P* = 0.009) (Figure 1L).

### Association of Th cells and their corresponding cytokines with depression

**Figure 2:**
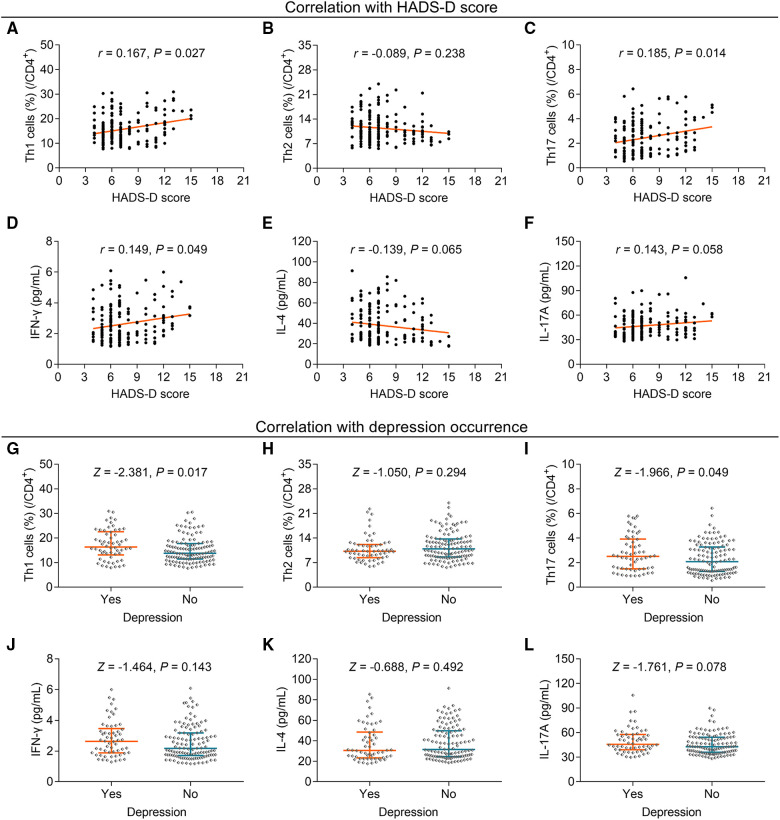
The association between Th1, Th2, and Th17 cells and depression in elderly gastric cancer patients. Association of Th1 (**A**), Th2 (**B**), Th17 cells (**C**), IFN-γ (**D**), IL-4 (**E**), and IL-17A (**F**) with HADS-D score; correlation of Th1 (**G**), Th2 (**H**), Th17 cells (**I**), IFN-γ (**J**), IL-4 (**K**), and IL-17A (**L**) with depression occurrence in elderly gastric cancer patients. Statistical methods: associations between continuous variables were analyzed using Spearman's rank correlation test; the levels of Th cells and cytokines between patients with or without anxiety, depression, and cognitive impairment were determined using the Wilcoxon rank-sum test.

Th1 cells (*P* = 0.027) (Figure 2A), but not Th2 cells (*P* = 0.238) (Figure 2B), were positively related to the HADS-D score; Th17 cells were positively associated with the HADS-D score (*P* = 0.014) (Figure 2C). Concerning the linkage between cytokines and the HADS-D score, only IFN-γ (*P* = 0.049) (Figure 2D), but not IL-4 (*P* = 0.065) (Figure 2E) or IL-17A (*P* = 0.058) (Figure 2F), was positively linked to the HADS-D score in elderly gastric cancer patients.

Subsequent analysis revealed that Th1 cells (*P* = 0.017) (Figure 2G), but not Th2 cells (*P* = 0.294) (Figure 2H), were enhanced in patients with depression compared with those without depression; Th17 cells were also increased in patients with depression compared to those without depression (*P* = 0.049) (Figure 2I). However, IFN-γ (*P* = 0.143) (Figure 2J), IL-4 (*P* = 0.492) (Figure 2K), and IL-17A (*P* = 0.078) (Figure 2L) levels did not differ between patients with and without depression.

### Relationship of Th cells and their corresponding cytokines with cognitive impairment

**Figure 3:**
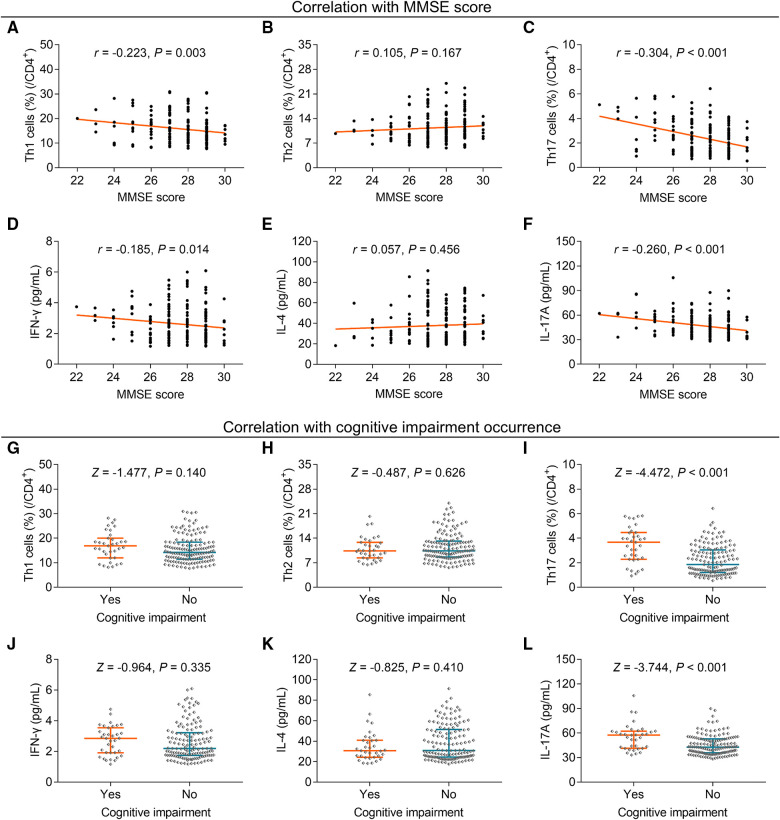
Correlation of Th1, Th2, and Th17 cells with cognitive impairment in elderly gastric cancer patients. Relationship between Th1 (**A**), Th2 (**B**), Th17 cells (**C**), IFN-γ (**D**), IL-4 (**E**), and IL-17A (**F**) and MMSE score; relationship between Th1 (**G**), Th2 (**H**), Th17 cells (**I**), IFN-γ (**J**), IL-4 (**K**), and IL-17A (**L**) with cognitive impairment occurrence in elderly gastric cancer patients. Statistical methods: associations between continuous variables were analyzed using Spearman's rank correlation test; the levels of Th cells and cytokines between patients with or without anxiety, depression, and cognitive impairment were determined using the Wilcoxon rank-sum test.

Th1 cells (*P* = 0.003) (Figure 3A), but not Th2 cells (*P* = 0.167) (Figure 3B), were negatively related to the MMSE score; concurrently, Th17 cells were inversely associated with the MMSE score (*P* < 0.001) (Figure 3C). Furthermore, a negative relationship was discovered between IFN-γ (*P* = 0.014) (Figure 3D), but not IL-4 (*P* = 0.456) (Figure 3E), and the MMSE score. Th17 cells were also inversely linked to the MMSE score (*P* < 0.001) (Figure 3F) in elderly gastric cancer patients.

Further analysis showed that Th1 cells (*P* = 0.140) (Figure 3G) and Th2 cells (*P* = 0.626) (Figure 3H) did not differ between patients with cognitive impairment and those without cognitive impairment, but Th17 cells were increased in patients with cognitive impairment compared to those without cognitive impairment (*P* < 0.001) (Figure 3I). Moreover, IFN-γ (*P* = 0.335) (Figure 3J) and IL-4 (*P* = 0.410) (Figure 3K) levels were similar between patients with and without cognitive impairment, but IL-17A was enhanced in patients with cognitive impairment compared to those without cognitive impairment (*P* < 0.001) (Figure 3L).

### Association of treatment information with anxiety, depression, and cognitive impairment

Neoadjuvant chemotherapy (*P* < 0.001), gastrectomy by open surgery (*P* = 0.022), postoperative complications (*P* < 0.001), and adjuvant chemotherapy (*P* < 0.001) were associated with an enhanced HADS-A score. Neoadjuvant chemotherapy (*P* = 0.001), postoperative complications (*P* < 0.001), and adjuvant chemotherapy (*P* = 0.002) were associated with an increased anxiety rate. In addition, postoperative complications (*P* < 0.001) and adjuvant chemotherapy (*P* = 0.016) were related to increased HADS-D scores. In addition, only postoperative complications were associated with a higher depression rate (*P* < 0.001). Moreover, postoperative complications were associated with elevated MMSE scores (*P* = 0.028), whereas treatment data were not related to cognitive impairment in elderly gastric cancer patients (all *P* > 0.05) (Table 4).

**Table 4:** Correlation of treatment information with anxiety, depression, and cognitive impairment.

Treatment information	Anxiety		Depression		Cognitive impairment	
	HADS-A score Mean ± SD	Anxiety rate No. (%)	HADS-D score Mean ± SD	Depression rate No. (%)	MMSE score Mean ± SD	Cognitive impairment rate No. (%)
Neoadjuvant chemotherapy						
No	7.2 ± 2.5	17 (25.8)	7.0 ± 2.4	17 (25.8)	27.5 ± 1.8	15 (22.7)
Yes	8.8 ± 2.8	57 (51.8)	7.7 ± 2.7	41 (37.3)	27.5 ± 1.5	20 (18.2)
*P* value	<0.001	0.001	0.083	0.116	0.961	0.465
Surgery type						
EMR/ESD	6.1 ± 1.0	0 (0.0)	6.4 ± 1.1	1 (12.5)	27.0 ± 1.2	2 (25.0)
Gastrectomy by open surgery	8.3 ± 2.8	73 (45.3)	7.5 ± 2.7	56 (34.8)	27.5 ± 1.5	31 (19.3)
Gastrectomy by laparoscope	6.4 ± 2.3	1 (14.3)	7.3 ± 3.5	1 (14.3)	27.6 ± 2.9	2 (28.6)
*P* value	0.022	0.459	0.483	0.857	0.637	0.898
Postoperative complication						
No	7.3 ± 2.2	30 (28.3)	6.8 ± 2.3	20 (18.9)	27.7 ± 1.5	16 (15.1)
Yes	9.5 ± 3.1	44 (62.9)	8.5 ± 2.9	38 (54.3)	27.2 ± 1.7	19 (27.1)
*P* value	<0.001	<0.001	<0.001	<0.001	0.028	0.050
Adjuvant chemotherapy						
No	6.2 ± 1.2	1 (6.3)	6.5 ± 1.4	3 (18.8)	26.8 ± 1.6	6 (37.5)
Yes	8.4 ± 2.8	73 (45.6)	7.6 ± 2.7	55 (34.4)	27.6 ± 1.6	29 (18.1)
*P* value	<0.001	0.002	0.016	0.205	0.061	0.094

HADS-A, Hospital Anxiety and Depression Scale-Anxiety; SD, standard deviation; HADS-D, Hospital Anxiety and Depression Scale-Depression; MMSE, Mini-Mental State Examination; EMR, endoscopic mucosal resection; ESD, endoscopic submucosal dissection. Statistical methods: the normally distributed continuous variables (HADS-A score, HADS-D score, and MMSE score) among patients with different treatment information were analyzed using Student's *t* test or one-way analysis of variance (ANOVA); the categorized variables (anxiety rate, depression rate, and cognitive impairment rate) among patients with different treatment information were analyzed using Chi-square test or linear by linear test.

### Correlation of treatment information with Th cells and cytokines

Postoperative complications were associated with increased Th1 cells (*P* = 0.019), but treatment information was not associated with Th2 cells and Th17 cells in elderly gastric cancer patients (all *P* > 0.05). Postoperative complications were related to decreased IL-4 levels in elderly gastric cancer patients (*P* = 0.029), whereas treatment information was not associated with IFN-γ or IL-17A (all *P* > 0.05) (Table 5).

**Table 5:** Correlation of treatment information with Th cells and cytokines.

Treatment information	Th1 cells (%) (/CD4^+^) Median (IQR)	Th2 cells (%) (/CD4^+^) Median (IQR)	Th17 cells (%) (/CD4^+^) Median (IQR)	IFN-γ (pg/ml) Median (IQR)	IL-4 (pg/ml) Median (IQR)	IL-17A (pg/ml) Median (IQR)
Neoadjuvant chemotherapy						
No	14.7 (11.8–19.0)	10.2 (8.7–12.6)	2.3 (1.4–3.8)	2.1 (1.7–3.6)	30.3 (24.6–48.0)	42.8 (34.2–54.4)
Yes	15.6 (11.4–18.5)	10.6 (8.5–13.4)	2.3 (1.3–3.2)	2.5 (1.8–3.2)	31.2 (24.4–51.0)	43.8 (37.9–55.6)
*P* value	0.787	0.552	0.310	0.412	0.954	0.114
Surgery type						
EMR/ESD	15.9 (9.3–20.9)	9.1 (8.5–11.5)	2.1 (1.1–3.6)	3.1 (1.5–3.8)	35.0 (25.4–54.4)	36.4 (34.3–45.2)
Gastrectomy by open surgery	14.9 (11.6–18.6)	10.5 (8.7–13.3)	2.3 (1.3–3.4)	2.2 (1.7–3.2)	30.8 (24.4–49.5)	43.4 (36.9–55.1)
Gastrectomy by laparoscope	14.8 (12.0–20.0)	9.7 (7.6–12.3)	3.5 (1.7–3.8)	2.3 (2.0–3.7)	37.6 (28.2–40.9)	46.7 (33.5–62.1)
*P* value	0.968	0.433	0.508	0.755	0.788	0.276
Postoperative complication						
No	13.7 (11.0–17.4)	10.7 (8.7–13.3)	2.3 (1.4–3.5)	2.2 (1.8–3.2)	34.3 (25.5–52.5)	42.9 (36.2–54.7)
Yes	16.0 (12.7–21.3)	10.1 (8.4–12.8)	2.2 (1.3–3.4)	2.6 (1.7–3.4)	28.1 (22.8–43.3)	46.6 (38.3–55.6)
*P* value	0.019	0.266	0.814	0.644	0.029	0.223
Adjuvant chemotherapy						
No	13.2 (8.7–17.8)	9.3 (8.5–12.3)	2.1 (1.1–3.4)	2.0 (1.5–3.6)	36.9 (28.4–54.4)	38.4 (34.3–58.1)
Yes	15.2 (11.8–18.8)	10.6 (8.6–13.3)	2.3 (1.3–3.4)	2.3 (1.8–3.2)	30.8 (24.4–49.3)	43.6 (36.9–55.0)
*P* value	0.115	0.424	0.601	0.433	0.198	0.342

Th, T helper; IFN, interferon; IL, interleukin; IQR, interquartile range; EMR, endoscopic mucosal resection; ESD, endoscopic submucosal dissection. Statistical methods: the skewed distributed continuous variables (Th cells and cytokines) among patients with different treatment information were analyzed using Wilcoxon rank-sum test or Kruskal-Wallis H rank-sum test.

### Forward stepwise multivariate logistic regression analyses for anxiety, depression, and cognitive impairment

Higher Th17 cells (/CD4^+^) [odds ratio (OR) = 1.573, *P* = 0.001], history of diabetes (yes vs. no) (OR = 3.574, *P* = 0.008), *Helicobacter pylori* infection (positive vs. negative) (OR = 2.964, *P* = 0.005), higher TNM stage (OR = 3.685, *P* < 0.001), and postoperative complications (yes vs. no) (OR = 4.858, *P* < 0.001) were all independently correlated with increased anxiety risk in elderly gastric cancer patients.

Residential location (urban vs. rural) (OR = 4.145, *P* = 0.036), history of diabetes (yes vs. no) (OR = 3.918, *P* = 0.004), *Helicobacter pylori* infection (positive vs. negative) (OR = 2.214, *P* < 0.037), and postoperative complications (yes vs. no) (OR = 5.625, *P* < 0.001) were all independently associated with increased depression risk in elderly gastric cancer patients.

Higher Th17 cells (/CD4^+^) (OR = 1.924, *P* < 0.001), higher IL-17A levels (OR = 1.039, *P* = 0.022), history of hyperlipidemia (yes vs. no) (OR = 2.870, *P* = 0.023), and postoperative complications (yes vs. no) (OR = 2.577, *P* = 0.044) were all independently related to increased cognitive impairment risk in elderly gastric cancer patients (Table 6).

**Table 6:** Forward stepwise multivariate logistic regression analyses for anxiety, depression, and cognitive impairment.

Items	*P* value	OR	95% CI	
			Lower	Upper
Forward stepwise multivariate logistic regression analyses for anxiety				
Higher Th17 cells (/CD4^+^)	0.001	1.573	1.198	2.066
History of diabetes (yes vs. no)	0.008	3.574	1.395	9.161
*Helicobacter pylori* infection (positive vs. negative)	0.005	2.964	1.396	6.293
Higher TNM stage	<0.001	3.685	1.861	7.297
Postoperative complications (yes vs. no)	<0.001	4.858	2.303	10.249
Forward stepwise multivariate logistic regression analyses for depression				
Residential location (urban vs. rural)	0.026	4.145	1.186	14.485
History of diabetes (yes vs. no)	0.004	3.918	1.550	9.903
*Helicobacter pylori* infection (positive vs. negative)	0.037	2.214	1.047	4.681
Postoperative complications (yes vs. no)	<0.001	5.625	2.684	11.787
Forward stepwise multivariate logistic regression analyses for cognitive impairment				
Higher Th17 cells (/CD4^+^)	<0.001	1.924	1.369	2.703
Higher IL-17A	0.022	1.039	1.005	1.073
History of hyperlipidemia (yes vs. no)	0.023	2.870	1.157	7.120
Postoperative complication (yes vs. no)	0.044	2.577	1.026	6.470

OR, odds ratio; CI, confidence interval; Th, T helper; TNM, tumor-node-metastasis; IL, interleukin. Statistical methods: factors related to anxiety, depression, and cognitive impairment were evaluated using a multivariate logistic regression model (all factors were included and then analyzed *via* the step-forward method).

### Comparison between elderly gastric cancer patients and elderly normal individuals

The HADS-A score (*P* < 0.001), anxiety rate (*P* = 0.001), anxiety severity (*P* < 0.001), HADS-D score (*P* < 0.001), depression rate (*P* = 0.003), depression severity (*P* = 0.003), Th1 cells (*P* = 0.034), and Th17 cells (*P* = 0.017) were increased in elderly gastric cancer patients compared to elderly normal individuals. In contrast, the MMSE score was decreased in elderly gastric cancer patients compared to elderly normal individuals (*P* = 0.036) (Supplementary Table S1).

## Discussion

The relationship of CD4^+^ T cells and their corresponding cytokines with anxiety and depression has been revealed by previous studies (17, 21). A previous study reported that CD4^+^ T cells are positively related to anxiety and depression in patients infected by the human immunodeficiency virus (21). Meanwhile, elevated IL-17 indicates anabatic anxiety and depression risks in NSCLC patients (17). However, the clinical impact of CD4^+^ T cells and their corresponding cytokines on anxiety, depression, and cognitive impairment in elderly gastric cancer patients is unknown. Notably, exploring this aspect may put a direction for further studies to explore the potential mechanism of CD4^+^ T cells for these mental and cognitive issues in elderly gastric cancer patients. In addition, the prevalence of anxiety, depression, and cognitive impairment in elderly gastric cancer patients deserves further confirmation. The current study showed that 42%, 33%, and 19.9% of elderly gastric cancer patients had anxiety, depression, and cognitive impairment, which was similar to some of the previous studies (22, 23). In addition, the prevalence of anxiety, depression, and cognitive impairment was increased in elderly gastric cancer patients compared to elderly normal individuals. This finding suggested that anxiety, depression, and cognitive impairment were serious issues in elderly gastric cancer patients. Additionally, it was also discovered that Th17 cells were positively linked to anxiety and slightly related to depression, while Th1 cells were positively linked with anxiety and depression to some extent. The potential arguments would be that (i) IL-17A and IFN-γ secreted by Th17 and Th1 cells might activate the kynurenine pathway leading to a decrease in serotonin levels, thereby causing anxiety and depression (24); (ii) Th17 cells participate in the gut-brain axis to facilitate stress responses (25), thus inducing anxiety and depression in elderly gastric cancer patients; (iii) increased Th1 and Th17-cellcell differentiation might lead to the recruitment of other immune cells and excessive microglial activation, which could further cause the occurrence of anxiety and depression (26–28); and (iv) increased Th1 and Th17 cells could boost the secretion of IFN-γ and IL-17A, which could enhance neuronal activation in the medial prefrontal cortex (29). Therefore, Th1 and Th17 cells were positively linked to anxiety and depression in elderly gastric cancer patients.

In addition to anxiety and depression, cognitive impairment is also a crucial issue in elderly gastric cancer patients (12). Several studies have reported the relationship of CD4^+^ T cells and cytokines with cognitive impairment (18, 30). For instance, a positive linkage is found between Th17 cells and cognitive impairment in Alzheimer's disease patients (30). Moreover, increased IL-4 could indicate aggravated cognitive impairment in breast cancer patients (18). This study discovered that Th17 cells were positively associated with cognitive impairment in elderly gastric cancer patients. This could be explained by the following: (i) Th17-cell-secreted IL-17A promoted SHSY5Y cell apoptosis, thus aggravating cognitive impairment (31); (ii) Th17-cell-secreted IL-17A would also exacerbate oxidative stress and neuroinflammation by activating the NF-*κ*B pathway, thereby intensifying cognitive impairment (32); (iii) the differentiation of Th cells might contribute to chronic neuroinflammation and further lead to cognitive impairment (33); and (iv) Th17 cells might also facilitate microglial activation, blood‒brain barrier breakdown, and antibody infiltration, thereby inducing cognitive impairment (34). As a result, Th17 and IL-17A were positively linked to cognitive impairment in elderly gastric cancer patients.

Currently, the treatments for elderly gastric cancer patients have made certain progress (4, 5). This study further evaluated the relationship of therapeutic strategies with anxiety, depression, cognitive impairment, CD4^+^ T cells, and corresponding inflammatory cytokines in elderly gastric cancer patients. Neoadjuvant and adjuvant chemotherapies were associated with increased anxiety in elderly gastric cancer patients. Possible reasons would be that neoadjuvant and adjuvant chemotherapies would cause a decline in quality of life (35, 36); thus, elderly gastric cancer patients receiving neoadjuvant or adjuvant chemotherapy were more likely to suffer from anxiety. In addition, postoperative complications were linked to elevated anxiety and depression in elderly gastric cancer patients. Potential explanations include complications, such as malnutrition, gastric perforation, leakage, pancreatic fistula, and gastrointestinal bleeding, that might negatively affect patients’ health or even lead to death (7–10, 37, 38); therefore, anxiety and depression were increased in elderly gastric cancer patients. Moreover, postoperative complications were associated with increased Th1 cells and reduced IL-4 in elderly gastric cancer patients. The explanation might be that postoperative complications cause inflammation. In addition, the dysregulation of Th1 and Th2 cells critically participates in the pathology and progression of inflammation (39). Therefore, an association was found between postoperative complications and Th1 and Th2 cell-secreted IL-4.

Several limitations might exist in this study: (1) To verify the relationship of Th cells with anxiety, depression, and cognitive impairment, this study also examined Th-cell-secreted cytokines. However, it should be noted that these cytokines might also be unavoidably affected by other cells; for example, IL17A might be secreted by neutrophil cells, thus causing some bias in the results. (2) The HADS score was assessed by elderly gastric cancer patients themselves; therefore, assessment bias might exist. (3) This study only enrolled elderly patients with resectable gastric cancer; thus, our findings in young, middle-aged adults or elderly patients with unresectable gastric cancer need to be explored by subsequent studies.

Conclusively, Th1 and Th17 cells could reflect anxiety, depression, and cognitive impairment risk to a certain extent in elderly gastric cancer patients, implying that these cells might participate in the pathology and progression of the abovementioned psychological and cognitive issues. Clinically, the findings of this study may provide a basis for better ameliorating mental health or improving the management of elderly gastric cancer patients. Nevertheless, further study is needed to investigate the detailed mechanism.

## Data Availability

The original contributions presented in the study are included in the article/**Supplementary Material**, further inquiries can be directed to the corresponding author/s.
