# Supplementary material for: Th1, Th2, and Th17 cells and their corresponding cytokines are associated with anxiety, depression, and cognitive impairment in elderly gastric cancer patients
Source: Front Surg. 2022 Oct 25;9:996680. doi: 10.3389/fsurg.2022.996680 (PMC9640774; doi:10.3389/fsurg.2022.996680)
Supplement: Supplementary file 1 [file Table7.docx]

**Supplementary table 1.** Comparison between elderly gastric cancer patients and elderly normal individuals.

| Items | Elderly normal individuals  (N = 30) | Elderly gastric cancer patients  (N = 176) | *P* value |
| --- | --- | --- | --- |
| HADS-A score, mean±SD | 4.5±2.5 | 8.2±2.8 | <0.001 |
| Anxiety rate, No. (%) | 3 (10.0) | 74 (42.0) | 0.001 |
| Anxiety severity, No. (%) |  |  | <0.001 |
| No | 27 (90.0) | 102 (58.0) |  |
| Mild | 3 (10.0) | 33 (18.8) |  |
| Moderate | 0 (0.0) | 36 (20.5) |  |
| Severe | 0 (0.0) | 5 (2.8) |  |
| HADS-D score, mean±SD | 4.5±2.3 | 7.5±2.6 | <0.001 |
| Depression rate, No. (%) | 2 (6.7) | 58 (33.0) | 0.003 |
| Depression severity, No. (%) |  |  | 0.003 |
| No | 28 (93.3) | 118 (67.0) |  |
| Mild | 2 (6.7) | 28 (15.9) |  |
| Moderate | 0 (0.0) | 27 (15.3) |  |
| Severe | 0 (0.0) | 3 (1.7) |  |
| MMSE score, mean±SD | 28.2±1.2 | 27.5±1.6 | 0.036 |
| Cognitive impairment rate, No. (%) | 2 (6.7) | 35 (19.9) | 0.081 |
| Cognitive impairment severity, No. (%) |  |  | 0.082 |
| No | 28 (93.3) | 141 (80.1) |  |
| Mild | 2 (6.7) | 35 (19.9) |  |
| Moderate | 0 (0.0) | 0 (0.0) |  |
| Severe | 0 (0.0) | 0 (0.0) |  |
| Th1 cells (%) (/CD4^+^), median (IQR) | 12.2 (10.3-16.7) | 15.0 (11.5-18.6) | 0.034 |
| Th2 cells (%) (/CD4^+^), median (IQR) | 11.8 (9.0-14.9) | 10.4 (8.6-13.1) | 0.194 |
| Th17 cells (%) (/CD4^+^), median (IQR) | 1.6 (1.0-2.5) | 2.3 (1.3-3.4) | 0.017 |

HADS-A, Hospital Anxiety and Depression Scale-Anxiety; SD, standard deviation; HADS-D, Hospital Anxiety and Depression Scale-Depression; MMSE, Mini-Mental State Examination; Th, T helper; IQR, interquartile range.

Statistical methods: the comparison between elderly gastric cancer patients and elderly normal individuals was assessed using Student's t test, the Mann‒Whitney U test, and the chi-square test.
